# Stability of Nuclear and Mitochondrial Reference Genes in Selected Tissues of the Ambrosia Beetle *Xylosandrus germanus*

**DOI:** 10.3390/insects12121125

**Published:** 2021-12-16

**Authors:** Nisha Patwa, Christopher M. Ranger, Maximilian Lehenberger, Peter H. Biedermann, Michael E. Reding

**Affiliations:** 1 Horticultural Insects Research Laboratory, USDA-Agricultural Research Service, 1680 Madison Ave., Wooster, OH 44691, USA; nisha.patwa@usda.gov (N.P.); mike.reding@usda.gov (M.E.R.); 2 Department of Biochemistry, Max Planck Institute for Chemical Ecology, Beutenberg Campus, Hans-Knoell-Strasse 8, D-07745 Jena, Germany; mlehenberger@ice.mpg.de; 3 Forest Entomology and Protection Research Group, University of Freiburg, D-79100 Freiburg im Breisgau, Germany; peter.biedermann@forento.uni-freiburg.de

**Keywords:** mycangium, fungus-farming beetle, symbiosis

## Abstract

**Simple Summary:**

The ambrosia beetle *Xylosandrus germanus* (Blandford) is a destructive wood-boring insect of horticultural tree crops. A fungal mutualist is cultivated within host trees that provides the sole source of nutrition for the larvae and adults of this beetle. Female *X. germanus* adults use a pouch-like structure (i.e., mycangium) to maintain and transport spores of their fungal mutualist. To facilitate future studies examining gene expression of *X. germanus*’ mycangium, the identification of stable genes unaffected by experimental treatments is needed to provide a standard reference during gene expression studies. Selected tissue types were dissected from laboratory-reared and field-collected specimens of the ambrosia beetle *X. germanus* to evaluate the stability of five reference genes, namely, 28S ribosomal RNA (*28S rRNA*), arginine kinase (*AK*), carbamoyl-phosphate synthetase 2-aspartate transcarbamylase-dihydroorotase (*CAD*), mitochondrial cytochrome oxidase 1 (*CO1*), and elongation factor-1α (*EF1*α). The reference genes *CO1* and *AK* were identified as primary and secondary reference genes. By contrast, *EF1*α was considered unsuitable for use as a reference gene during gene expression studies with *X. germanus*. These results will aid in normalizing the expression of target genes during studies with *X. germanus.*

**Abstract:**

The fungus-farming ambrosia beetle *Xylosandrus germanus* (Blandford) uses a pouch-like structure (i.e., mycangium) to transport spores of its nutritional fungal mutualist. Our current study sought to identify reference genes necessary for future transcriptome analyses aimed at characterizing gene expression within the mycangium. Complementary DNA was synthesized using selected tissue types from laboratory-reared and field-collected *X. germanus* consisting of the whole body, head + thorax, deflated or inflated mycangium + scutellum, inflated mycangium, and thorax + abdomen. Quantitative reverse-transcription PCR reactions were performed using primers for 28S ribosomal RNA (*28S rRNA*), arginine kinase (*AK*), carbamoyl-phosphate synthetase 2-aspartate transcarbamylase-dihydroorotase (*CAD*), mitochondrial cytochrome oxidase 1 (*CO1*), and elongation factor-1α (*EF1*α). Reference gene stability was analyzed using GeNorm, NormFinder, BestKeeper, ΔCt, and a comprehensive final ranking by RefFinder. The gene *CO1* was identified as the primary reference gene since it was generally ranked in first or second position among the tissue types containing the mycangium. Reference gene *AK* was identified as a secondary reference gene. In contrast, *EF1*α was generally ranked in the last or penultimate place. Identification of two stable reference genes will aid in normalizing the expression of target genes for subsequent gene expression studies of *X. germanus’* mycangium.

## 1. Introduction

**Figure 1:**
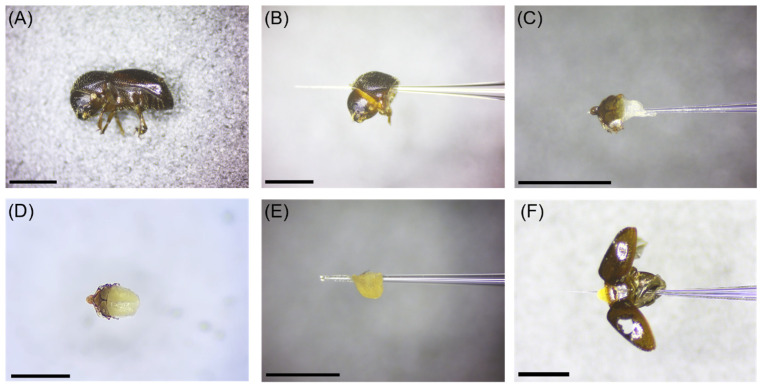
Selected tissue types dissected from laboratory-reared (LR) and field-collected (FC) specimens of *Xylosandrus germanus* consisting of (**A**) whole body of LR and FC female specimens, (**B**) head + pronotum of LR and FC specimens, (**C**) deflated mycangium + scutellum from LR specimens only, (**D**) inflated mycangium + scutellum of LR and FC specimens, (**E**) inflated mycangium from LR and FC specimens, and (**F**) inflated mycangium + scutellum + thorax (excluding the pronotum) + abdomen from LR and FC specimens. Scale bar in each figure represents 1.0 mm.

Ambrosia beetles (Coleoptera: Curculionidae: Scolytinae) are fungus-farming insects that burrow into host tissues for cultivating fungal mutualists on which the larvae and adults must feed [1]. Infestations can lead to branch dieback and the death of trees growing in ornamental nurseries and tree fruit and nut orchards [1,2,3,4]. Ambrosia beetles are recognized among the most successful biological invaders; in particular, 30 exotic species in the tribe Xyleborini are established in North America [5,6]. *Xylosandrus germanus* (Blandford) is a xyleborine ambrosia beetle from Asia and is established in numerous U.S. states and the Canadian provinces of British Columbia, Nova Scotia, Ontario, and Québec [6]. Adult females are about 2.0 mm in length (Figure 1A) [2].

*Ambrosiella grosmanniae* C. Mayers, McNew & T.C. Harrington is the nutritional fungal mutualist of *X. germanus* [7]. As an obligate fungal mutualist, *A. grosmanniae* relies on female beetles for dispersal and is not free-living in the absence of *X. germanus* [8]. A membranous pouch-like structure (i.e., mycangium, singular form of mycangia) set within the mesothorax enables adult female *X. germanus* to transport spores of *A. grosmanniae* [9,10]. Unlike mandibular mycangia (like in *Xyleborus* spp.) that support multiple dominant fungal symbionts, which may not all be beneficial to the beetles (i.e., mutualists), mesothoracic mycangia support one type of dominant fungal symbiont specific and beneficial to the ambrosia beetle host [11,12]. *Ambrosiella grosmanniae* has only been isolated in association with *X. germanus* and not other ambrosia beetles [7,12].

While still in their natal host tree gallery, the mycangium of freshly-molted adult *X. germanus* is deflated and absent of a fungal mass [10]. By contrast, mature adult *X. germanus* that have dispersed from their natal gallery possess mycangia that are partially or fully inflated with spores of *A. grosmanniae* [10]. In studies on the mesothoracic mycangia of *Cnestus mutilatus* (Blandford), Stone et al. [13] demonstrated Type 1 secretory cells present in the outer secretory layer containing electron-dense material that is released into the lumen of the mycangium. Secretory gland cells releasing fatty acids, amino acids, and/or proteins into the mycangium have been proposed to stimulate the growth of ambrosia beetle fungal mutualists and perhaps inhibit antagonistic fungi [13,14,15,16]. Micro-computed tomography is providing an insight into the structure and function of ambrosia beetle mycangia [10,17], but the chemical basis by which mycangia support mutualist growth remains unknown [18].

The objective of our current study was to identify reference genes or “housekeeping genes” exhibiting consistent expression in selected tissue types of laboratory-reared and field-collected *X. germanus.* Since reference genes have consistent expression regardless of experimental conditions, they are used as internal controls during gene expression studies to normalize variability associated with experimental conditions [19,20]. Specifically, the stability of five references genes (*28S rRNA*, *AK*, *CAD*, *CO1*, *EF1α*) were analyzed in different body tissues of *X. germanus*. Two stable reference genes identified during this study will be used to normalize quantitative reverse-transcription PCR (RT-qPCR) data during upcoming transcriptome analyses of *X. germanus’* mycangium.

## 2. Materials and Methods

### 2.1. Insect Specimens and Tissue Types

Adult female *X. germanus* were used for comparing reference gene expression in selected tissue types from laboratory-reared (LR) specimens and natural, field-collected (FC) specimens. Specifically, we selected the following six tissue types from laboratory-reared specimens: (1) whole body, (2) head + pronotum without scutellum, (3) deflated mycangium + scutellum, (4) inflated mycangium + scutellum, (5) inflated mycangium without scutellum, and (6) deflated/inflated mycangium + thorax (without pronotum) + abdomen from laboratory-reared *X. germanus* (Figure 1). The following five tissue types were collected from field-collected specimens: (1) whole body, (2) head + pronotum without scutellum, (3) inflated mycangium + scutellum, (4) inflated mycangium without scutellum, and (5) inflated mycangium + thorax (without pronotum) + abdomen from laboratory-reared *X. germanus* (Figure 1). The deflated mycangium + scutellum tissue type was not obtained from field-collected adults because dispersing field-collected specimens were only found to possess a partially or fully inflated mycangium [10]]. Recently eclosed adult female xyleborine ambrosia beetles in their natal galleries possess a deflated mycangium that has not yet become full of fungal symbiont spores, while dispersing adults that have left their natal gallery are characterized by an inflated mycangium full of fungal symbiont spores as described by Li et al. [10]. Specimens of *X. germanus* were identified using morphological features as described by Gomez et al. [5].

Laboratory-reared adult female *X. germanus* were reared from an agar-based substrate containing sawdust from the tree *Cercis canadensis* L. [21,22]. Multiple field-collected *X. germanus* were used to initiate the colonies. Specimens from the F_1_–F_4_ generations were used during the tissue dissections. Adult females collected from the rearing tubes were held in Petri dishes with moistened paper towel at room temperature until tissue dissections were initiated. To obtain deflated mycangia that had not yet become full of spores of the fungal mutualist *A. grosmanniae*, some of the laboratory-reared beetles were immediately used for dissections upon their emergence as adults from the rearing substrate. To obtain inflated mycangia that were enlarged due to spores of *A. grosmanniae*, some of the laboratory-reared beetles were held in the Petri dishes for 5 days and then used for dissections.

Field-collected adult female *X.* *germanus* were obtained on multiple dates in May through June 2020 during peak spring flight of overwintered adults from a mixed hardwood forested area at the Ohio Agricultural Research and Development Center in Wayne Co., Wooster, OH, USA (40°45′40.85″ N, 81°51′14.71″ W) using a bottle trap baited with an ethanol lure (65 mg/d at 30 °C; AgBio, Inc., Westminster, CO, USA) [23]. A moistened paper towel rolled into a tube was placed in the bottom collection vessel of the trap to maintain living ambrosia beetle specimens until they were transferred (within 24 h) from the traps to parafilm-sealed Petri dishes containing moistened filter paper [24]. Field-collected specimens were held at 22 °C for 24 to 48 h until tissue dissections were initiated under laboratory conditions as described below.

For each biological replicate, dissected tissues were pooled according to tissue type from individual *X. germanus* laboratory-reared and field-collected specimens for whole body (*n* = 20 FC and *n* = 20 LR beetles per biological replicate), head + pronotum (*n* = 20 FC and *n* = 20 LR beetles per biological replicate), deflated mycangium + scutellum (*n* = 75 LR beetles per biological replicate), inflated mycangium + scutellum (*n* = 75 LR and n = 75 FC beetles per biological replicate), inflated mycangium alone (*n* = 75 LR and *n* = 75 FC beetles per biological replicate), and inflated mycangium + scutellum + thorax (excluding the pronotum) + abdomen (*n* = 20 FC and *n* = 20 LR beetles per biological replicate) (Figure 1a–f). Three biological replicates for each of the tissue types from laboratory-reared and field-collected specimens were used for this study. Three technical replicates were analyzed from each biological replicate for analysis.

Whole body, head + pronotum, and mycangium + scutellum + thorax (excluding the pronotum) + abdominal tissues were initially homogenized to a powder using a chilled mortar and pestle with liquid N_2_ followed by the addition of TRIzol reagent (Invitrogen, Carlsbad, CA, USA). The heterogenous solution was then transferred to 2 mL tubes with 0.5 mm ceramic beads and macerated using an Omni Bead Ruptor Elite (OMNI International, Inc., Kennesaw, GA, USA) programmed to 8 cycles of 5 m/s for 20 s with 10 min incubation on ice every 4 cycles. Similarly, dissected tissues of inflated and deflated mycangium with and without scutellum in TRIzol reagent were also placed in individual 2 mL tubes with 0.5 mm ceramic beads and macerated using a Bead Ruptor Elite and the aforementioned program. After centrifugation at 12,000× *g* for 5 min at 4 °C, the supernatant was transferred to a new 2 mL Eppendorf tube for subsequent RNA isolation.

### 2.2. RNA Isolation, Reverse Transcription, and Primer Design

Total RNA was isolated from the selected tissues using TRIzol reagent (Invitrogen, Carlsbad, CA, USA) by following TRIzol method. Integrity and concentration of the RNA was verified using a 1% formaldehyde agarose gel and measuring absorbance at 260/280 nm to confirm 1.8–2.2 ODU using a BioSpectrometer Nanodrop (Eppendorf, Hamburg, Germany). Total RNA (1000 µg) was purified using DNase I (Sigma-Aldrich, St. Louis, MO, USA) and then used to synthesize first strand cDNA using RevertAid First Strand cDNA Synthesis Kit (Thermo Scientific, Waltham, MA, USA). Based on Dole et al. [25], five reference genes were selected as candidate genes from *X. germanus*, namely, 28S ribosomal RNA (*28S rRNA*; GenBank Accession: GU808598), arginine kinase (*AK*; GU808674), carbamoyl-phosphate synthetase 2-aspartate transcarbamylase-dihydroorotase (*CAD*; GU808635), mitochondrial cytochrome oxidase 1 (*CO1*; GU808714), and elongation factor-1α (*EF1*α; GU808751). Sequences of these five genes were obtained from the National Center for Biotechnology Information (NCBI) and translated to amino acids by ExPASy tool [26]. Primers were subsequently designed from the coding sequence using Primer3 [27] (Table 1). Primers were synthesized by Integrated DNA Technologies (Coralville, IA, USA).

### 2.3. Real-Time PCR

Synthesized cDNA was 5-fold serially-diluted and 1 µL was then combined with 25 µM of each primer (forward and reverse), nuclease free ddH_2_O, and 10 µL of 1× SsoFast EvaGreen Supermix in 20 µL of reaction mixture (Bio-Rad Laboratories, Inc., Hercules, CA, USA). Reactions were amplified using CFX96 Touch Real-Time Thermal Cycler qPCR System (Bio-Rad Laboratories, Inc., Hercules, CA, USA) with the following conditions: beginning cycle of 30 s at 95 °C for enzyme activation, 40 cycles of 10 s at 95 °C for denaturation, and 20 s at 53 °C for annealing and extension. Three technical replicates were analyzed for each of the three biological replicates prepared from each of the tissue types from laboratory-reared and field-collected specimens to obtain the quantification cycle (Cq) values. For each forward and reverse primer pair, amplifications included a no-template control to ensure contaminants and primer-dimer formations were not present. Melt curves were generated for each reaction from 65–95 °C with an increment of 0.5 °C to ensure the presence of a single peak to rule out non-specific product and dimer formations. The RT-qPCR efficiency for each candidate reference gene was calculated using a slope analysis and linear regression model. A standard curve method and 5-fold serial dilutions of cDNA were used to calculate the correlation coefficient (R^2^) to validate each primer. The resulting RT-qPCR amplification efficiency (E) was calculated using: E (%) = (10^(−1/slope)^ − 1) × 100 [28]. Gel electrophoresis (1%) was used to confirm the amplicon size for each reference gene and to ensure the absence of non-specific bands.

### 2.4. Stability and Statistical Analysis of Candidate Reference Genes

Cq values resulting from the RT-qPCR reactions were used for comparing the stability of reference genes. Specifically, the Cq values were used to assess reference gene stability for the selected tissue types for laboratory-reared and field-collected specimens using the following Microsoft Excel-based computational algorithms: GeNorm [29], NormFinder [30], BestKeeper [31], and the comparative ΔCt method [32].

The statistical algorithm GeNorm measures reference gene stability using the geometric average and pairwise variation of all candidate reference genes to produce an M-score (M) whereby the most stable gene exhibits the lowest M value [29]. GeNorm ultimately identifies the best pair of genes instead of the single best gene for accurate normalization of RT-qPCR data [29]. An analysis of variance (ANOVA) approach is used by NormFinder to measure intra- and inter-group variability of the candidate genes to produce an overall stability value (SV) whereby a lower SV score represents higher stability [30]. Pearson’s correlation coefficient, standard deviation (SD), and percentage covariance (CV) of cycle threshold (Ct) values are considered by BestKeeper to create a ranked index of each reference gene whereby a low SD value denotes stability [31]. The comparative ΔCt algorithm considers the relative expression value of reference genes in pairs and ranks gene stability according to reproducibility of gene expression whereby a lower score denotes higher stability [32].

Since the aforementioned algorithms might produce different rankings among the reference genes, raw Cq values were entered into the web-based tool RefFinder (RefFinder. Available online: http://www.heartcure.com.au/reffinder/# (accessed on 3 May 2021)) to integrate all four statistical algorithms and assign a final comprehensive ranking of reference gene stability based on the geometric means [33]. Lower geometric means generated by RefFinder denote higher stability.

## 3. Results and Discussion

### 3.1. Primer Specificity and Efficiency of Reference Genes

**Figure 2:**
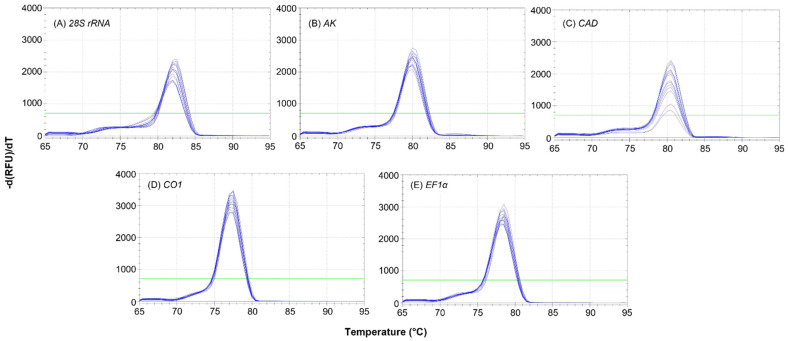
Melt curves demonstrating the presence of a single peak to rule out non-specific products following RT-qPCR reactions for the following primers (**A**) *28S rRNA*, 28S ribosomal RNA; (**B**) *AK*, arginine kinase; (**C**) *CAD*, carbamoyl-phosphate synthetase 2-aspartate transcarbamylase-dihydroorotase, (**D**) *CO1*, cytochrome oxidase subunit 1, and (**E**) *EF1α*, elongation factor 1-α. Horizontal green line represents baseline threshold.

The five selected primers resulted in specific and efficient RT-qPCR reactions. A single peak evident in each melt curve analysis confirmed the primer specificity during PCR amplification reactions for all five of the candidate reference genes (Figure 2).

There were no indications of non-specific amplification products or primer-dimer formations. Additionally, no peaks were detected from the no-template control reactions as demonstrated by flat baselines in the melt curve analyses (Figure 2). Reaction efficiency (E) ranged from 99.0 to 108.7% and correlation coefficient (R^2^) values ranged from 0.998 to 0.999 across the five primers (Table 1). The PCR reactions for the five primers produced single amplicon bands of expected size for each gene when analyzed by gel electrophoresis (data not presented).

### 3.2. Expression Profiling of Reference Genes

**Figure 3:**
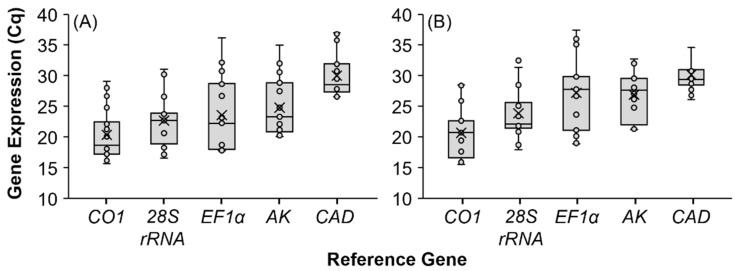
Expression in quantification cycle (Cq) values of five reference genes (*28S*, 28S ribosomal RNA; *AK*, arginine kinase; *CAD*, carbamoyl-phosphate synthetase 2-aspartate transcarbamylase-dihydroorotase; *CO1*, cytochrome oxidase subunit 1; *EF1α*, elongation factor 1-α) as represented by pooled Cq values obtained for whole body and all dissected tissue types from (**A**) laboratory-reared and (**B**) field-collected samples of *X. germanus*. Three technical replicates were analyzed for each of the three biological replicates that were prepared from each of the tissue types from laboratory-reared and field-collected specimens to generate the quantification cycle (Cq) values. The resulting Cq values from the technical replicates were then averaged and the mean values were used to prepare the boxplots for each reference gene. Data include six pooled tissue types for laboratory-reared specimens and five pooled tissue types for field-collected specimens.

Expression profiles for the five reference genes were determined using their corresponding primers and RT-qPCR in tissues from laboratory-reared and field-collected *X. germanus* (Figure 3). Reference gene expression levels are presented as cycle quantification (Cq) values, which are defined as the number of RT-qPCR cycles required for the florescence of the PCR product to pass a threshold detection level. The Cq value represents an inverse relationship whereby elevated gene expression corresponds to a low Cq value.

The same pattern was documented in expression stability of the selected reference genes in tissues from laboratory-reared and field-collected *X. germanus*. Reference gene *CO1* exhibited comparatively low Cq values in tissues from laboratory-reared and field-collected *X. germanus*, which corresponds to high expression levels. Conversely, *CAD* showed comparatively high Cq values, thereby indicating low expression levels in laboratory-reared and field-collected *X. germanus*. Moderate Cq values were exhibited by *EF1α* (Figure 3).

A reference gene exhibiting a comparatively moderate Cq expression level is preferred because a high or low expression level can introduce variability [34,35,36]. For instance, Kyre et al. [36] selected the ribosomal protein *rps18* as a reference gene for RNAi experiments involving the southern pine beetle, *Dendroctonus frontalis* Zimm. because *rps18* was moderately expressed according to Cq values and was ranked in the first position by two of four algorithms and comprehensively by RefFinder. We did not observe a close association between gene expression according to Cq values and the ranking of reference gene stability based on the four algorithms and comprehensively according to RefFinder. For instance, *EF1*α was moderately expressed according to Cq values in our analyses of gene expression profiling, but *EF1*α was consistently ranked by the comprehensive RefFinder analysis in the last or penultimate place among the five reference genes for tissues containing mycangia. As described by Ruiz-Villalba et al. [37], interpreting Cq values is no longer advisable because Cq values can vary between samples, runs, and instruments. Thus, we only considered the rankings from the four individual algorithms (i.e., GeNorm, NormFinder, BestKeeper, ΔCt) and the comprehensive RefFinder analysis to interpret reference gene stability.

### 3.3. Stability Analysis of Reference Genes

The Cq values generated from the RT-qPCR reactions allowed for ranking reference gene stability using GeNorm, NormFinder, BestKeeper, and ΔCt (Table 2). Results from these algorithms were then analyzed using RefFinder to provide a comprehensive final ranking of gene stability for laboratory-reared and field-collected specimens of *X. germanus* in the following six tissue types: whole body, head + pronotum, deflated mycangium + scutellum, inflated mycangium + scutellum, inflated mycangium, and inflated mycangium + scutellum + thorax (excluding the pronotum) + abdomen (Table 2). We avoided interpreting Cq values as part of our current study following the recommendation by Ruiz-Villalba et al. [37] and, instead, considered the rankings from the four algorithms GeNorm, NormFinder, BestKeeper, and ΔCt followed by the final RefFinder ranking.

Overall, *CO1* was considered the most stable reference gene for tissues types containing mycangia (i.e., whole body, head + pronotum, scutellum + deflated and inflated mycangium, and inflated mycangium) (Table 2). On a scale of 1–5 (with 1 being the best), the mean final RefFinder ranking of *CO1* for all tissue types containing mycangia was 1.8 for both laboratory-reared and field-collected specimens. The mean final rankings of *28S rRNA*, *AK*, and *CAD* were 2.8, 3, and 3.2 for laboratory-reared specimens, and 3.8, 3, and 1.8 for field-collected specimens, respectively. In contrast, the mean final ranking of the reference gene *EF1α* for all tissue types containing mycangia were 4.2 and 4.8 for laboratory-reared and field-collected specimens, respectively.

Specifically, the reference gene *CO1* was ranked in the first position by the comprehensive RefFinder analysis in whole body tissues, and head + pronotum tissues, from laboratory-reared and field-collected *X. germanus* (Table 2). The reference gene *CO1* was assigned to the second position in the final ranking by RefFinder for inflated mycangium + scutellum from both laboratory-reared and field-collected *X. germanus*. The reference gene *CO1* was placed in the second and third position for inflated mycangium from laboratory-reared and field-collected specimens, respectively. A ranking of third position was assigned to *CO1* for deflated mycangium + scutellum.

The ranking of reference gene stability in the abdominal tissues of laboratory-reared and field-collected beetles resulted in a different pattern compared to the other tissue types. In particular, the reference gene *EF1α* was placed in a final ranking of first and third position for thorax + abdominal tissues from laboratory-reared and field-collected *X. germanus*, respectively (Table 2). A final ranking of first and second position was also assigned to the reference gene *AK* for thorax + abdominal tissues from field-collected and laboratory-reared specimens, respectively.

We selected *CO1* as the primary reference gene for subsequent transcriptome analyses involving the mycangium from *X. germanus* since it was generally ranked in first or second place by RefFinder for the dissected tissues containing mycangia. Similarly, Ridgeway and Timm [38] found *CO1* to have comparatively stable expression in the whole body tissue of *Thaumatotibia leucotreta* (Meyrick) by the three algorithms GeNorm, NormFinder, and BestKeeper. The reference genes *28S rRNA*, *AK*, or *CAD* could be used as secondary reference genes in support of *CO1* since using two or more reference genes can improve the reliability of results during gene expression studies [34,35,36,37,39]. The reference gene *AK* might be the most suitable choice since previous studies have found unstable gene expression of *28S rRNA* and *CAD* in insects [40,41,42]. Our current study identified *EF1*α as the most unstable reference gene for tissue types containing mycangia.

## 4. Conclusions

Identifying reference genes with stable and consistent expression is necessary to normalize the expression of target genes when using RT-qPCR [34]. Since a “universal” reference gene has not been identified [34], it is important to assess reference gene stability within selected insect tissues and according to experimental treatments. Based on phylogenetic studies by Dole et al. [25] that sequenced nuclear and mitochondrial genes in *X. germanus* and other Scolytinae, we examined the expression of five reference genes consisting of two protein-encoding genes (*AK* and *CAD*), a ribosomal-encoding gene (*28S rRNA*), a mitochondrial gene (*CO1*), and a nuclear protein-encoding gene elongation factor (*EF1*α). Specific treatments were not imposed on *X. germanus* as part of our current study, but the experimental design did incorporate laboratory-reared and field-collected beetles as two different sources of specimens for assessing reference gene stability. In preparation for tissue-specific transcriptome analyses, we characterized reference gene stability in the whole body, head + pronotum, deflated mycangium + scutellum, inflated mycangium + scutellum, inflated mycangium alone, and inflated mycangium + scutellum + thorax (excluding the pronotum) + abdomen. Based on rankings from the four individual algorithms (i.e., GeNorm, NormFinder, BestKeeper, ΔCt) and the comprehensive RefFinder analysis, we interpreted *CO1* and *AK* as the two most stable reference genes for subsequent gene expression studies focused on the mycangium of *X. germanus*.

## Figures and Tables

**Table 1:** RT-qPCR primers used for five reference genes in *X. germanus*.

Gene Symboland Name	Primer Sequence (5′–3′)	Length (bp)	Efficiency (%)	R^2^ Regression
*28S rRNA* 28S Ribosomal RNA	F: ACTCCTTGGTCCGTGTTTCA R: CTCTGGTGACTGTTGGCGA	120	099.0	0.998 y = −3.346 + 31.033
*AK* Arginine kinase	F: ACAAGTCTACCGTCGTCTGG R: GTTGGTTGGGCAGAAAGTGA	103	105.8	0.999 y = −3.190 + 30.798
*CAD* carbamoyl-phosphate synthetase 2-aspartate transcarbamylase-dihydroorotase	F: GAACCACCGCCATAAACGTT R: TGGACAGTCGGGCATTAACT	120	108.7	0.998 y = −3.129 + 38.058
*CO1* Cytochrome oxidase subunit 1	F: TTCCTCCTGCTAAAACTGGC R: CCTCAATCCTTGGAGCAATCA	150	104.0	0.999 y = −3.229 + 25.483
*EF1α* Elongation factor-1 alpha	F: CCAACCAGAAATAGGCACGA R: CCACCGAACCACCCTACAG	119	108.3	0.999 y = −3.138 + 28.178

**Table 2:** Stability and rank values of reference genes in tissue samples of laboratory-reared (LR) and field-collected (FC) *X. germanus* as determined using the algorithms GeNorm, NormFinder, BestKeeper, ΔCt and a comprehensive final ranking by RefFinder. Rank represents a scale of 1–5 with 1 being best.

Tissue	Source	Gene	GeNorm		NormFinder		BestKeeper		ΔCt		RefFinder	
			Stability	Rank	Stability	Rank	Stability	Rank	Stability	Rank	Stability	Final Rank
Whole Body	LR	*CO1*	0.137	1	0.069	1	0.24	2	0.40	1	1.19	1
		*EF1α*	0.137	1	0.069	2	0.16	1	0.40	2	1.41	2
		*CAD*	0.215	2	0.109	3	0.31	4	0.44	3	3.22	3
		*AK*	0.305	3	0.516	4	0.30	3	0.58	4	3.72	4
		*28S*	0.544	4	0.886	5	0.91	5	0.90	5	5.00	5
	FC	*CO1*	0.136	1	0.068	1	0.16	2	0.78	1	1.19	1
		*AK*	0.136	1	0.389	2	0.14	1	0.81	2	1.41	2
		*CAD*	0.870	3	0.908	3	1.00	4	1.06	3	3.46	3
		*EF1α*	0.380	2	1.051	4	0.52	3	1.12	4	3.46	4
		*28S*	0.985	4	1.088	5	1.12	5	1.16	5	5.00	5
Head + Pronotum	LR	*CO1*	0.784	2	0.468	1	0.84	3	1.19	1	1.73	1
		*CAD*	0.181	1	1.068	3	0.21	1	1.32	3	1.73	2
		*28S*	0.181	1	1.264	4	0.26	2	1.42	4	2.38	3
		*AK*	1.148	3	0.759	2	1.62	4	1.32	2	2.83	4
		*EF1α*	1.411	5	1.710	5	2.19	5	1.81	5	5.00	5
	FC	*CO1*	1.308	2	0.849	1	1.83	3	1.85	1	1.73	1
		*CAD*	0.279	1	1.708	3	0.32	2	2.09	2	1.86	2
		*28S*	0.279	1	2.095	4	0.12	1	2.28	4	2.00	3
		*AK*	1.958	3	1.565	2	2.60	4	2.12	3	3.13	4
		*EF1α*	2.165	4	2.295	5	3.17	5	2.48	5	5.00	5
Inflated Mycangium + Scutellum	LR	*AK*	0.359	1	0.179	1	2.43	3	1.47	1	1.32	1
		*CO1*	0.359	1	0.610	2	2.71	4	1.56	2	2.00	2
		*28S*	1.546	3	1.251	3	1.25	2	1.98	3	2.91	3
		*CAD*	1.985	4	2.512	5	0.55	1	2.64	5	3.34	4
		*EF1α*	0.887	2	2.136	4	3.57	5	2.27	4	3.94	5
	FC	*CAD*	0.315	1	0.989	2	0.13	1	1.21	2	1.41	1
		*CO1*	0.527	2	0.296	1	0.60	3	1.01	1	1.73	2
		*28S*	0.315	1	1.092	4	0.21	2	1.26	3	2.21	3
		*AK*	1.090	3	1.063	3	1.70	4	1.35	4	3.72	4
		*EF1α*	1.275	4	1.456	5	1.90	5	1.55	5	5.00	5
Inflated Mycangium	LR	*28S*	0.494	1	1.467	3	0.36	1	1.98	2	1.57	1
		*CO1*	1.161	2	0.702	1	0.84	3	1.71	1	1.73	2
		*CAD*	0.494	1	1.854	4	0.50	2	2.16	3	2.21	3
		*EF1α*	1.696	3	1.435	2	1.92	4	2.24	4	3.36	4
		*AK*	2.216	4	2.798	5	2.66	5	3.00	5	5.00	5
	FC	*CAD*	0.371	1	0.185	1	0.40	2	5.32	1	1.19	1
		*AK*	0.371	1	0.185	2	0.30	1	5.38	2	1.41	2
		*CO1*	0.832	2	0.338	3	1.13	3	5.43	3	3.00	3
		*28S*	1.029	3	3.140	4	1.52	4	5.81	4	4.00	4
		*EF1α*	8.160	4	18.846	5	16.11	5	18.86	5	5.00	5
Deflated Mycangium + Scutellum	LR	*AK*	0.361	1	0.181	1	0.69	3	0.75	1	1.32	1
		*28S*	0.361	1	0.588	2	0.38	1	0.85	2	1.41	2
		*CO1*	0.560	2	0.902	4	0.45	2	1.04	4	3.13	3
		*CAD*	0.844	3	0.723	3	1.33	4	0.94	3	3.46	4
		*EF1α*	0.927	4	0.934	5	1.37	5	1.05	5	5.00	5
Mycangium + Thorax (without pronotum) + Scutellum + Abdomen ^1^	LR	*EF1α*	0.121	1	0.207	1	0.38	3	0.52	1	1.32	1
		*AK*	0.121	1	0.375	3	0.31	1	0.55	2	1.57	2
		*CO1*	0.138	2	0.379	4	0.32	2	0.56	3	2.91	3
		*CAD*	0.331	3	0.243	2	0.77	4	0.64	4	3.36	4
		*28S*	0.707	4	1.253	5	1.52	5	1.27	5	5.00	5
	FC	*AK*	0.118	1	0.047	1	0.38	2	0.65	1	1.19	1
		*CAD*	0.118	1	0.144	2	0.40	3	0.66	2	1.86	2
		*EF1α*	0.309	2	0.188	3	0.29	1	0.69	3	2.28	3
		*28S*	0.650	3	1.053	4	0.81	5	1.15	4	4.23	4
		*CO1*	0.869	4	1.117	5	0.64	4	1.20	5	4.73	5

^1^ A combination of deflated and inflated mycangium were used for LR mycangium + thorax (without pronotum) + scutellum + abdomen, but inflated mycangium were used for FC mycangium + thorax (without pronotum) + scutellum + abdomen.

## Data Availability

The data presented in this study are readily available on request from the corresponding author.

## References

[1] Hulcr J., Stelinski L.L. (2017). The ambrosia symbiosis: From evolutionary ecology to practical management. Annu. Rev. Entomol..

[2] Ranger C.M., Reding M.E., Schultz P.B., Oliver J.B., Frank S.D., Addesso K.M., Chong J.H., Sampson B., Werle C., Gill S. (2016). Biology, ecology, and management of nonnative ambrosia beetles (Coleoptera: Curculionidae: Scolytinae) in ornamental plant nurseries. J. Integr. Pest Manag..

[3] Agnello A.M., Breth D.I., Tee E.M., Cox K.D., Villani S.M., Ayer K.M., Wallis A.E., Donahue D.J., Combs D.B., Davis A.E. (2017). *Xylosandrus germanus* (Coleoptera: Curculionidae: Scolytinae) occurrence, fungal associations, and management trials in New York apple orchards. J. Econ. Entomol..

[4] Monterrosa A., Acebes A.L., Blaauw B., Joseph S.V. (2021). Effects of trap, and ethanol lure type and age on attraction of ambrosia beetles (Coleoptera: Curculionidae). J. Econ. Entomol..

[5] Gomez D.F., Rabaglia R.J., Fairbanks K.E., Hulcr J. (2018). North American Xyleborini north of Mexico: A review and key to genera and species (Coleoptera, Curculionidae, Scolytinae). ZooKeys.

[6] CABI (Commonwealth Agricultural Bureaux International) Invasive Species Compendium (2019). Xylosandrus germanus (Black Timber Bark Beetle).

[7] Mayers C.G., McNew D.L., Harrington T.C., Roeper R.A., Fraedrich S.W., Biedermann P.H., Castrillo L.A., Reed S.E. (2015). Three genera in the Ceratocystidaceae are the respective symbionts of three independent lineages of ambrosia beetles with large, complex mycangia. Fungal Biol..

[8] Harrington T.C., Aghayeva D.N., Fraedrich S. (2010). New combinations in *Raffaelea*, *Ambrosiella*, and *Hyalorhinocladiella*, and four new species from the redbay ambrosia beetle, *Xyleborus glabratus*. Mycotaxon.

[9] Hulcr J., Cognato A.I. (2010). Repeated evolution of crop theft in fungus-farming ambrosia beetles. Evol. Intern. J. Org. Evol..

[10] Li Y., Ruan Y.Y., Stanley E.L., Skelton J., Hulcr J. (2019). Plasticity of mycangia in *Xylosandrus* ambrosia beetles. Insect Sci..

[11] Kostovcik M., Bateman C.C., Kolarik M., Stelinski L.L., Jordal B.H., Hulcr J. (2015). The ambrosia symbiosis is specific in some species and promiscuous in others: Evidence from community pyrosequencing. ISME J..

[12] Ito M., Kajimura H. (2017). Landscape-scale genetic differentiation of a mycangial fungus associated with the ambrosia beetle, *Xylosandrus germanus* (Blandford) (Curculionidae: Scolytinae) in Japan. Ecol. Evol..

[13] Stone W.D., Nebeker T.E., Monroe W.A., MacGown J.A. (2007). Ultrastructure of the mesonotal mycangium of *Xylosandrus mutilatus* (Coleoptera: Curculionidae). Can. J. Zool..

[14] Schneider I.A., Rudinsky J.A. (1969). Mycetangial glands and their seasonal changes in *Gnathotrichus retusus* and *G. sulcatus*. Ann. Entomol. Soc. Am..

[15] Schneider I., Rudinsky J.A. (1969). Anatomical and histological changes in internal organs of adult *Trypodendron lineatum*, *Gnalhotrichus retusus*, and *G. sulcatus* (Coleoptera: Scolytidae). Ann. Entomol. Soc. Am..

[16] Norris D.M., Batra L.R. (1979). The Mutualistic Fungi of the Xyleborini beetles. Insect-Fungus Symbiosis: Nutrition, Mutualism, and Commensalism.

[17] Spahr E., Kasson M.T., Kijimoto T. (2020). Micro-computed tomography permits enhanced visualization of mycangia across development and between sexes in *Euwallacea* ambrosia beetles. PLoS ONE.

[18] Joseph R., Keyhani N.O. (2021). Fungal mutualisms and pathosystems: Life and death in the ambrosia beetle mycangia. Appl. Microbiol. Biotechnol..

[19] Hruz T., Wyss M., Docquier M., Pfaffl M.W., Masanetz S., Borghi L., Verbrugghe P., Kalaydjieva L., Bleuler S., Laule O. (2011). RefGenes: Identification of reliable and condition specific reference genes for RT-qPCR data normalization. BMC Genom..

[20] Kozera B., Rapacz M. (2013). Reference genes in real-time PCR. J. Appl. Gen..

[21] Castrillo L.A., Griggs M.H., Vandenberg J.D. (2012). Brood production by *Xylosandrus germanus* (Coleoptera: Curculionidae) and growth of its fungal symbiont on artificial diet based on sawdust of different tree species. Environ. Entomol..

[22] Ranger C.M., Biedermann P.H., Phuntumart V., Beligala G.U., Ghosh S., Palmquist D.E., Mueller R., Barnett J., Schultz P.B., Reding M.E. (2018). Symbiont selection via alcohol benefits fungus farming by ambrosia beetles. Proc. Natl. Acad. Sci. USA.

[23] Ranger C.M., Reding M.E., Persad A.B., Herms D.A. (2010). Ability of stress-related volatiles to attract and induce attacks by *Xylosandrus germanus* and other ambrosia beetles. Agric. For. Entomol..

[24] Ranger C.M., Schultz P.B., Frank S.D., Chong J.H., Reding M.E. (2015). Non-native ambrosia beetles as opportunistic exploiters of living but weakened trees. PLoS ONE.

[25] Dole S.A., Jordal B.H., Cognato A.I. (2010). Polyphyly of *Xylosandrus* Reitter inferred from nuclear and mitochondrial genes (Coleoptera: Curculionidae: Scolytinae). Mol. Phylogenet. Evol..

[26] Artimo P., Jonnalagedda M., Arnold K., Baratin D., Csardi G., deCastro E., Duvaud S., Flegel V., Fortier A., Gasteiger E. (2012). ExPASy: SIB bioinformatics resource portal. Nucleic Acids Res..

[27] Rozen S., Skaletsky H. (2000). Primer3 on the WWW for general users and for biologist programmers. Methods Mol. Biol..

[28] Radonić A., Thulke S., Mackay I.M., Landt O., Siegert W., Nitsche A. (2004). Guideline to reference gene selection for quantitative real-time PCR. Biochem. Biophys. Res. Commun..

[29] Vandesompele J., de Preter K., Pattyn F., Poppe B., van Roy N., de Paepe A., Speleman F. (2002). Accurate normalization of real-time quantitative RT-PCR data by geometric averaging of multiple internal control genes. Genome Biol..

[30] Andersen C.L., Jensen J.L., Orntoft T.F. (2004). Normalization of real-time quantitative reverse transcription-PCR data: A model-based variance estimation approach to identify genes suited for normalization, applied to bladder and colon cancer data sets. Cancer Res..

[31] Pfaffl M., Tichopad A., Prgomet C., Neuvians T. (2004). Determination of stable housekeeping genes, differentially regulated target genes and sample integrity: BestKeeper—Excel-based tool using pair-wise correlations. Biotechnol. Lett..

[32] Silver N., Best S., Jiang J., Thein S.L. (2006). Selection of housekeeping genes for gene expression studies in human reticulocytes using real-time PCR. BMC Mol. Biol..

[33] De Spiegelaere W., Dern-Wieloch J., Weigel R., Schumacher V., Schorle H., Nettersheim D., Bergmann M., Brehm R., Kliesch S., Vandekerckhove L. (2015). Reference gene validation for RT-qPCR, a note on different available software packages. PLoS ONE.

[34] Lü J., Chen S., Guo M., Ye C., Qiu B., Wu J., Yang C., Pan H. (2018). Selection and validation of reference genes for RT-qPCR analysis of the ladybird beetle *Henosepilachna vigintioctomaculata*. Front. Physiol..

[35] Barros Rodrigues T., Khajuria C., Wang H., Matz N., Cardoso D.C., Valicente F.H., Zhou X., Siegfried B. (2014). Validation of reference housekeeping genes for gene expression studies in western corn rootworm (*Diabrotica virgifera virgifera*). PLoS ONE.

[36] Kyre B.R., Rodrigues T.B., Rieske L.K. (2019). RNA interference and validation of reference genes for gene expression analyses using qPCR in southern pine beetle, *Dendroctonus frontalis*. Sci. Rep..

[37] Ruiz-Villalba A., Ruijter J.M., van den Hoff M.J. (2021). Use and misuse of Cq in qPCR data analysis and reporting. Life.

[38] Ridgeway J.A., Timm A.E. (2015). Reference gene selection for quantitative real-time PCR normalization in larvae of three species of *Grapholitini* (Lepidoptera: Tortricidae). PLoS ONE.

[39] Sang W., He L., Wang X.P., Zhu-Salzman K., Lei C.L. (2015). Evaluation of reference genes for RT-qPCR in *Tribolium castaneum* (Coleoptera: Tenebrionidae) under UVB stress. Environ. Entomol..

[40] Xue J.L., Salem T.Z., Turney C.M., Cheng X.W. (2010). Strategy of the use of 28S rRNA as a housekeeping gene in real-time quantitative PCR analysis of gene transcription in insect cells infected by viruses. J. Virol. Methods.

[41] Salem T.Z., Allam W.R., Thiem S.M. (2014). Verifying the stability of selected genes for normalization in Q PCR experiments of *Spodoptera frugiperda* cells during AcMNPV infection. PLoS ONE.

[42] Chang Y.W., Chen J.Y., Lu M.X., Gao Y., Tian Z.H., Gong W.R., Zhu W., Du Y.Z. (2017). Selection and validation of reference genes for quantitative real-time PCR analysis under different experimental conditions in the leafminer *Liriomyza trifolii* (Diptera: Agromyzidae). PLoS ONE.

